# Postpartum Early EMDR therapy Intervention (PERCEIVE) study for women after a traumatic birth experience: study protocol for a randomized controlled trial

**DOI:** 10.1186/s13063-021-05545-6

**Published:** 2021-09-06

**Authors:** Y. M. G. A. Hendrix, K. S. M. van Dongen, A. de Jongh, M. G. van Pampus

**Affiliations:** 1 Department of Obstetrics and Gynaecology, OLVG, Amsterdam, the Netherlands; 2 Academic Centre for Dentistry Amsterdam (ACTA), University of Amsterdam and VU Amsterdam, Amsterdam, the Netherlands; 3 Research Department, PSYTREC, Bilthoven, the Netherlands; 4 School of Health Sciences, Salford University, Manchester, UK; 5 Institute of Health and Society, University of Worcester, Worcester, UK; 6 School of Psychology, Queen’s University, Belfast, Northern Ireland

**Keywords:** Eye movement desensitization and reprocessing therapy, EMDR, Posttraumatic stress disorder, PTSD, Trauma, Childbirth, Delivery, Obstetrics, Postpartum

## Abstract

**Background:**

Up to 33% of women develop symptoms of posttraumatic stress disorder (PTSD) after a traumatic birth experience. Negative and traumatic childbirth experiences can also lead to fear of childbirth, avoiding or negatively influencing a subsequent pregnancy, mother-infant bonding problems, problems with breastfeeding, depression and reduced quality of life. For PTSD in general, eye movement desensitization and reprocessing (EMDR) therapy has proven to be effective. However, little is known about the preventive effects of early intervention EMDR therapy in women after a traumatic birth experience. The purpose of this study is to determine the effectiveness of early intervention EMDR therapy in preventing PTSD and reducing PTSD symptoms in women with a traumatic birth experience.

**Methods:**

The PERCEIVE study is a randomized controlled trial. Women suffering from the consequences of a traumatic birth experience will be randomly allocated at maximum 14 days postpartum to either EMDR therapy or ‘care-as-usual’. Patients in the EMDR group receive two sessions of therapy between 14 (T0) and 35 days postpartum. All participants will be assessed at T0 and at 9 weeks postpartum (T1). At T1, all participants will undergo a CAPS-5 interview about the presence and severity of PTSD symptoms. The primary outcome measure is the severity of PTSD symptoms, whereas the secondary outcomes pertain to fear of childbirth, mother-infant bonding, breastfeeding, depression and quality of life. The study will be conducted at a large city hospital and at multiple midwifery practices in Amsterdam, the Netherlands.

**Discussion:**

It is to be expected that the results of this study will provide more insight about the safety and effectiveness of early intervention EMDR therapy in the prevention and reduction of PTSD (symptoms) in women with a traumatic birth experience.

**Trial registration:**

Netherlands Trial Register NL73231.000.20. Registered on 21 August 2020.

## Background

According to recent meta-analyses, 3–4% of all women develop PTSD following childbirth [1, 2] while up to 33% of women experience symptoms of PTSD [3, 4]. Risk factors for a traumatic birth experience are diverse. Prevalence of traumatic birth experiences has been found to be higher among women with unexpected interventions during labour and delivery, such as unplanned caesarean section or vacuum-assisted delivery [3, 5]. However, medically uncomplicated deliveries may also be perceived as traumatic [3, 5–8]. A Dutch retrospective survey analysing the perception of over 2000 women with a self-reported traumatic birth experience showed that lack, or loss, of control (54.6%), and fear for their baby’s health or life (49.9%), followed by high intensity of pain or physical discomfort (47.4%) attributed significantly to the traumatic aspect of this experience [9]. Other reported risk factors include a history of psychiatric illness, previous trauma, fear of childbirth (FoC) and preeclampsia [2, 5].

According to the Dutch Society of Obstetrics and Gynaecology (NVOG) guidelines on birth-related PTSD (symptoms) [10] and the National Institute for Health and Care Excellence (NICE) treatment guidelines [11], a traumatic birth experience is defined as ‘the (subjective) experience and interpretation of a woman with or without satisfying diagnostic criteria of the Diagnostic and Statistical Manual of Mental Disorder (DSM-) 5 for PTSD’ [10, 11]. DSM-5 criteria of PTSD include symptoms of re-experiencing, avoidance and numbing, negative cognitions and mood and hyperarousal [12]. Symptoms may resolve naturally, but in some cases may lead to a chronic mental health condition [13]. To meet the diagnostic criteria for PTSD, symptoms must last longer than 1 month and result in significant dysfunction. However, due to similarity with physiological symptoms associated with a major life event, such as becoming a parent, PTSD (symptoms) are often not well recognized. Additionally, PTSD can be confused with postpartum depression because of a diagnostic overlap in symptoms such as negative cognitions, or co-occurrence of both disorders [13].

Besides PTSD, exposure to a negative or traumatic childbirth frequently results in fear of childbirth (FoC), avoiding a subsequent pregnancy, reduced quality of life (QoL), problems with breastfeeding and depression [5, 14–17]. A birth-related trauma causing distress during a subsequent pregnancy has also been found to be related to both maternal and foetal negative outcomes, such as avoiding prenatal care, demanding a planned caesarean section, and preterm birth [18–21]. Furthermore, traumatic birth experiences may negatively influence the mother-infant bonding with studies showing that children from parents with PTSD express a more avoidant attachment style, caused by feelings of rejection and anger towards the infant [22, 23]. Mother-infant bonding and early attachment proves essential for the infants’ future self-esteem and resilience, emotion regulation and their ability to form close relationships [24, 25]. Conversely, studies have shown that the presence of PTSD in parents is associated with the development of psychopathology and higher rates of anxiety and behavioural problems in their offspring [14, 26]. Moreover, it may impair early cognitive development of the infant [27]. To this end, it is conceivable that preventing PTSD, or alleviating PTSD symptoms, reduces the likelihood of mother-infant bonding difficulties, thereby exerting a positive effect on both the mother and a ‘butterfly effect’ on the infant to create a healthier adult in later life [26]. Hence, research that would identify a short-term and cost-effective postpartum intervention is important.

Regarding the treatment of PTSD in general, meta-analyses and treatment guidelines recommend eye movement desensitization and reprocessing (EMDR) therapy as one of the first-line therapies for this mental health condition [8, 28–30]. Additionally, encouraging results have been reported with early EMDR interventions after traumatic events to reduce PTSD-related symptoms [31, 32]. Although the definition of early intervention varies between guidelines, a timeframe of treatment within 3 months after the traumatic incident is mostly considered to be early intervention [32]. However, a recent review on EMDR therapy studies concluded that ‘research is needed to evaluate prevention of PTSD, with clinician-administered diagnostic measures (e.g., CAPS-5) administered to treated and nontreated individuals’ (p. 245) [33]. Recently, a randomized controlled pilot study was performed among women with a traumatic birth experience aimed to study the effect of EMDR therapy on symptoms of PTSD directly postpartum in comparison with treatment as usual, which consisted of standard psychological supportive care. The proportion of asymptomatic participants was significantly higher in the EMDR therapy group at 6 weeks postpartum (78.9% vs 39.4%, *p* = 0.020) although this effect was no longer statistically significant at 12 weeks postpartum (89.5% vs 66.7%, *p* = 0.124) [34]. Since this was a pilot study, sample size was small (*n* = 37) which could account for the lack of differences between groups at 12 weeks postpartum. This encouraging result calls for replication through a randomized controlled trial [33]. Finally, it is important to note that data on the safety of early interventions following recent trauma, particularly regarding exposure to adverse birth events, are limited [35, 36].

## Methods and design

### Aim

The main purpose of the study is to determine the safety and effectiveness of ‘early intervention EMDR’ in preventing PTSD or reducing symptoms of PTSD at 9 weeks postpartum in women with a traumatic birth experience, and to compare these results with care as usual (CAU) in women who experienced their delivery as a traumatic event. Safety is defined as the absence of all of the following adverse events: increased suicidal ideation, serious self-injurious behaviour or crisis contacts for any of the aforementioned reasons [37]. It is hypothesized that early intervention EMDR therapy is safe and that women who receive early intervention EMDR will report significantly less PTSD (symptoms) 9 weeks after the delivery compared to women who receive no treatment. Furthermore, our secondary objectives are to determine the effects of early intervention EMDR therapy on FoC, QoL, mother-infant bonding (MIB), breastfeeding and depression. It is hypothesized that early intervention EMDR will significantly reduce FoC, improve QoL and MIB, increase the success of breastfeeding and prevent or reduce depressive symptoms as compared to CAU.

### Study design

**Fig. 1:**
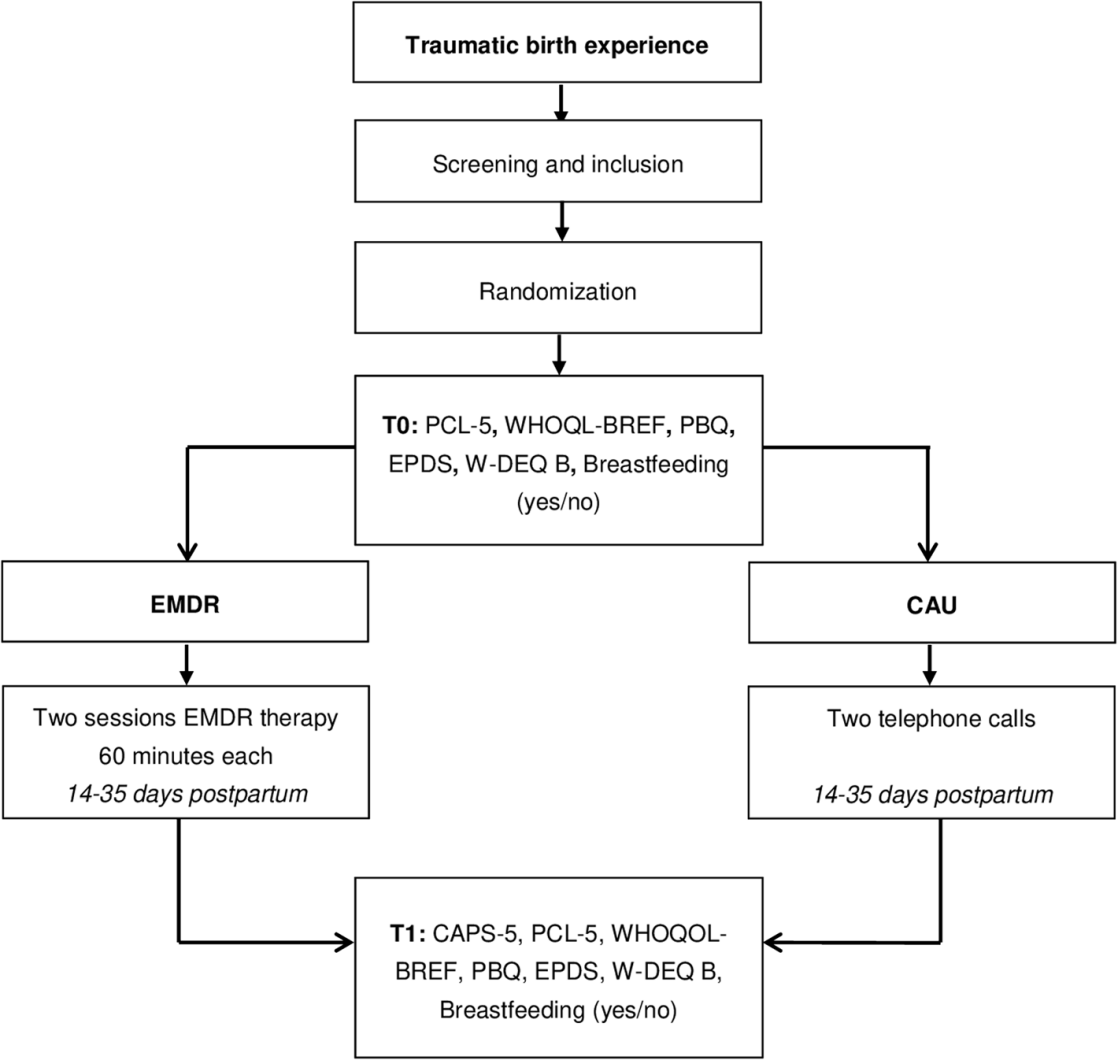
Flow chart

**Fig. 2:**
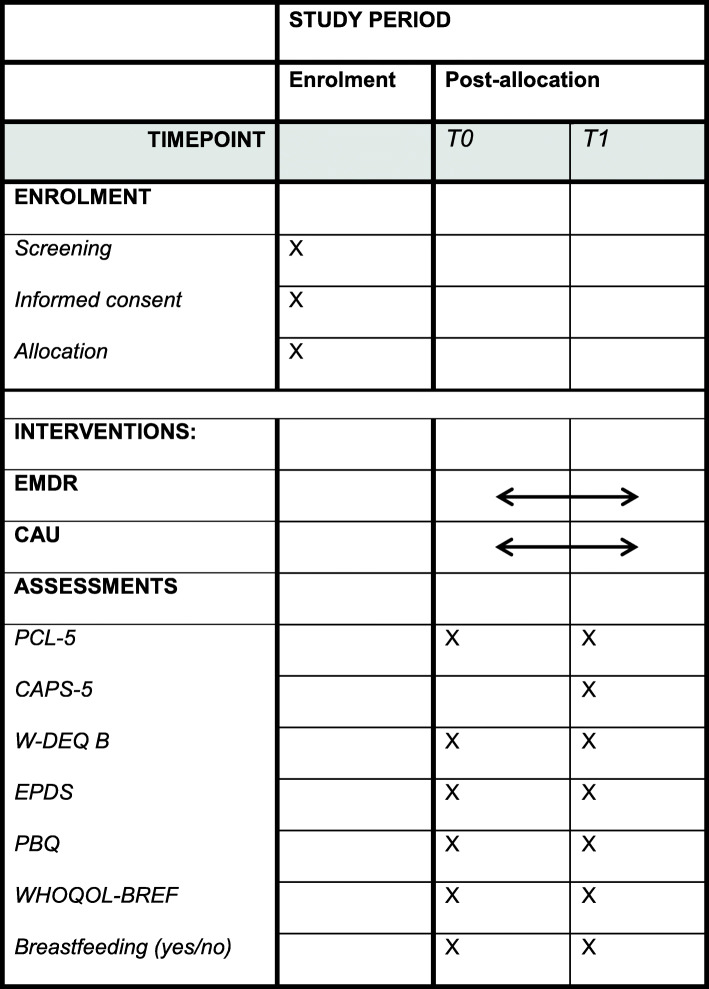
Enrolment and assessments over time

The PERCEIVE study will use a randomized controlled experimental design. A total of 216 women with traumatic birth experience will be recruited within 14 days postpartum and randomized to either the early EMDR intervention or CAU. Women will be asked to participate by their midwife or by the researchers at the end of the postpartum midwifery care period, which is 8 to 10 days postpartum. To ensure sufficient time to read the patient information and provide written consent, randomization has been set to take place at 14 days postpartum. Patients in the early EMDR therapy group will receive two sessions of EMDR therapy between 14 and 35 days postpartum as it is expected that a time frame of 3 weeks (21 days) is feasible to ensure adherence of participants to the protocol. Patients in the CAU group will receive no EMDR treatment but will receive two telephone calls during the study period. The two groups will be compared on a number of outcome variables before (T0 = 2 weeks) and post-treatment (T1 = 9 weeks; see Figs. 1 and 2). The study endpoint (T1) is at 9 weeks postpartum to establish the possible presence of PTSD accurately as defined by the DSM-5 criteria for PTSD.

Women will be recruited in a large hospital and several community midwifery practices in the Amsterdam area, the Netherlands. We expect that the study duration will encompass 2 years from start inclusions to end inclusions. The study protocol is approved by The Medical Research Ethics Committee of the OLVG Hospital and registered with trialregister.nl (reference no. NL73231.000.20).

### Patients

#### Inclusion criteria

Women less than 14 days postpartum who report a traumatic birth experience will be asked to participate in this study. Medical deliveries as well as women who had a delivery supervised by a primary care midwife from community midwifery practices in Amsterdam will be included. Furthermore, they must understand the Dutch language.

#### Exclusion criteria

Exclusion criteria include age less than 18 years old, birth trauma related to a previous birth, recent diagnosis of a psychiatric disorder, recent or current worsening of symptoms of a previously diagnosed psychiatric disorder requiring treatment or a recent history of a suicide attempt, that is, less than 3 months prior to the beginning of the study.

### Procedures and interventions

When women agree to participate, they will receive a home visit from the researchers to ensure women acknowledged the information and to fill out an informed consent. After screening and providing informed consent, participants will be randomly allocated to either the EMDR therapy or the CAU group within 14 days postpartum (see Fig. 1). Randomization will be on a 1:1 basis by block randomization with random block sized of two, four or six, performed using Castor EDC [38]. After completing the baseline assessments (T0), participants will be informed in which group they have been randomized.

#### The EMDR therapy group

Participants in the EMDR therapy group will receive two treatment sessions between 14 and 35 days postpartum, consisting of 60 min each [39]. The EMDR therapy sessions will be conducted in an out-hospital clinic by trained psychologists who have completed both the basic and advanced EMDR therapy training course accredited by the Dutch National EMDR Association (www.emdr.nl) and have at least 1 year of experience with providing EMDR therapy. When the participant or therapist is not able to be physically present because of the COVID-19 epidemic, therapy sessions may, by exception, be conducted digitally. Digital psychological interventions during COVID-19 are supported by the American Psychiatric Association and the British Psychological Society [40, 41].

The essence of EMDR therapy is that the therapist aims to reduce the vividness and emotionality of trauma memories by asking the patient to recall the trauma memory while simultaneously making eye movements [42, 43]. The EMDR therapy will be implemented with the use of rapid deployment of sets of eye movements offered by fingers or using a light bar [39]. In case of any adverse events during the study period, the EMDR therapist will report this to the researcher and set up an individual plan with regard to the wishes and needs of the participant.

#### The CAU group

The CAU group will receive care as provided currently which means no treatment for their traumatic birth experience. However, participants in this group will receive two telephone calls between 14 and 35 days postpartum to monitor the course of symptoms regarding their traumatic birth experience. These conversations will be conducted by the researcher. Preferably, no professional psychological treatment will be given during the study period as to not bias the results. Therefore, when a participant is stable but wishes to receive EMDR treatment, she will be asked to wait for therapy until she has filled in the last questionnaires and the Clinician-Administered PTSD Scale of DSM-5 (CAPS-5) interview is conducted at the end of the study period (T1). When symptoms of PTSD significantly worsen during the study period, women will be referred to their general practitioner who can then refer the patient to a psychologist or psychiatrist when needed. When professional psychological help regarding the birth trauma is started within the study period, this will be registered and reported as a deviation of the protocol.

### Assessments

#### Screening

After giving birth, all maternity women will receive a flyer with information about the study, regardless of the type of delivery or the presence of birth complications. Eight to 10 days postpartum, all women will be asked the following question: ‘How did you experience the delivery of your baby?’ Depending on the place of the delivery, this question will be asked either by the researcher or by their own midwife. Women will be considered eligible for the study if the answer includes the word ‘trauma’ or ‘traumatic’, or when the patient indicates to suffer from symptoms appropriate to PTSD.

#### Safety

Safety of early intervention EMDR will be monitored by the therapist. The occurrence of all adverse events, which is defined as increased suicidal ideation, serious self-injurious behaviour or contacts with healthcare providers in case of mental health crisis, will be reported to the researcher. Safety of participants in the CAU group will be monitored by the researchers. Based on regular phone calls, it will be estimated whether additional interventions regarding their psychological symptoms are indicated. For both groups, the researcher will register the adverse event and the general practitioner will be informed to take appropriate measures to ensure the safety of the participants.

#### Effectiveness

Other outcome variables will be assessed using multiple questionnaires. After randomization, but prior to the treatment (T0), both groups will receive the PTSD checklist for DSM-5 (PCL-5) [44], World Health Organization Quality of Life Questionnaire-BREF (WHOQOL-BREF) [45], Postpartum Bonding Questionnaire (PBQ) [46], Wijma Delivery Expectancy/Experience Questionnaire version B (W-DEQ B) [47], Early Postpartum Depression Scale (EPDS) [48] and questions on breastfeeding (see Figs. 1 and 2). While the experiences of the women are leading in this study, the results of patients’ questionnaire scores will not influence the inclusion and randomization process. Questionnaires will be sent through Castor with a unique ID code. If participants do not have access to a computer, the questionnaires will be provided on paper with a return envelope.

Nine weeks postpartum (at T1), each participant in both groups will undergo a CAPS-5 interview [49] and will receive the same questionnaires as at T0. To establish whether the time criterion (i.e. criterion F) of the DSM-5 criteria for PTSD has been met, the CAPS-5 interview will be conducted 4 weeks after the last EMDR therapy session in the intervention group, which corresponds to 9 weeks postpartum. The CAPS interview will be conducted by an independent person who is blinded to the randomization and who has received official training in assessment of the CAPS as to not bias the results. The CAPS version regarding symptoms in the last month will be used.

### Instruments

The following measurement instruments will be used:

#### Posttraumatic Stress Disorder Checklist (PCL-5)

The PCL-5 is a 20-item self-report questionnaire corresponding to the symptoms in the DSM-5 and is rated from 0 (not at all) to 4 (extremely) [44]. Total symptom severity scores ranging from 0 to 80 can be obtained by summing the scores for each of the 20 items. A total score ≥31 has been found suggestive for a probable PTSD in the English version [50]. Additionally, symptom clusters following the different DSM-5 criteria can be analysed separately [51]. A previous study found strong internal consistency, test-retest reliability, convergent validity and discriminant validity of the PCL-5 [52]. The reported clinically significant change on a DSM–IV version of PCL was found to be 10 points [53]. For the PCL-5, this clinically significant change has not been set yet.

#### Clinician-Administered PTSD Scale for DSM-5 (CAPS-5)

PTSD diagnosis as well as PTSD symptom severity will be assessed by using the Dutch version of the CAPS-5 [49, 54]. The CAPS-5 is a structured clinical interview that enables standardized DSM-5 PTSD diagnosis based on symptom severity scores. The interview consists of 20 questions regarding PTSD symptom severity (B–E items) and several questions concerning other DSM-5 criteria. PTSD diagnosis can be made by following the DSM-5 diagnostic rule, which requires the A criterion (exposure to (imminent) dead, severe injury or sexual violence), ≥1 B item (questions 1–5), ≥1 C item (questions 6–7), ≥2 D items (questions 8–14), ≥2 E items (questions 15–20), the F criterion (duration ≥1 month), G criterion (causing significant suffering or disability) and H criterion (symptoms are not caused by another medical condition or substance use). Questions regarding the B-E criteria are rated from 0 (‘absent’) to 4 (‘disabling’). Symptoms rated ≥2 are included in the calculation for the diagnosis. To assess whether criterion A was met during birth, specific questions will be used according to the work of Alcorn et al. [55]. The exact questions are as follows: (1) ‘Did you feel that your life or your baby’s life was threatened during or after birth?’ (2) ‘Did you think that you or your baby might die?’ (3) ‘Did you experience an actual injury or threat of serious injury around the time of birth?’ (4) ‘Did your baby experience an actual injury or threat of serious injury around the time or birth?’ [55]. High validity and reliability of the CAPS-5 have been found [49, 56].

#### World Health Organization Quality of Life assessment (WHOQOL-BREF) [45]

Quality of life is measured by using the Dutch version of the WHOQOL-BREF. It contains 26 items covering four domains: physical health, psychological health, social relationships and environment. Domain scores can be transformed to a score ranging from 0 to 100 with higher scores reflecting better quality of life. There is no cut-off point to demonstrate better or worse quality of life in this particular population. It has been validated for this specific population [57].

#### Postpartum Bonding Questionnaire (PBQ)

Mother to infant bonding is assessed by using the PBQ [46]. It consists of 25 statements, each followed by a 6-point Likert scale ranging from ‘Always’ (0) to ‘Never’ (5) with a range of 0–125 with higher scores reflecting a problematic mother-to-infant bond. Good validity and reliability of the Dutch version of the PBQ have been found [58]. To analyse the effects of PTSD symptom severity on the PBQ, only the total score will be used since later validation studies have not been able to replicate its factor structure. A score of 26 or higher is suggesting for some kind of bonding disorder, whereas a score of 40 or higher indicates severe bonding disorders [59].

#### Wijma Delivery Experience Questionnaire version B (W-DEQ B)

The W-DEQ B is used to measure postpartum fear of childbirth. The W-DEQ is a 33-item self-report questionnaire assessing FoC during pregnancy (version A), and after delivery (version B) in terms of the woman’s cognitive appraisal of childbirth [47]. It was designed as a monofactorial scale, all 33 items being scored on a 6-point scale leading to a sum score between 0 and 165, with a higher score equalling more FoC. High reliability and promising validity of the W-DEQ B have been found [47].

#### Edinburgh Postnatal Depression Scale (EPDS)

The Dutch version of the EPDS is a self-reporting questionnaire designed to assess pregnancy and postpartum depression; it is composed of 10 items scored on a 4-point Likert scale. Higher scores reflect a greater level of depression severity with a cut-off score of ≥13 to screen positive for depression [60]. The Dutch version of EPDS is a widely used measure with good psychometric characteristics [48].

#### Breastfeeding

Women will be asked whether they had the intention to breastfeed and whether they are breastfeeding at T0 and T1 by using ‘yes’ and ‘no’ questions.

### Demographics

Questions on the socio-demographic background will be included consisting of questions on country of birth, educational level, partner status, previous or current psychologic/psychiatric treatment, previous and or current medical treatment, medication use, fertility treatment, planned pregnancy, care in pregnancy, complications during pregnancy, following a pregnancy course and preference for mode of delivery (vaginal birth or caesarean section) prior to the actual mode of delivery. Furthermore, medical data on the delivery itself and demographics will be extracted from the electronic patient file or the birth report as made by the community midwife.

### Sample size calculation

Based on a mean difference between two treatment arms of at least 10 points on the PCL-5 scale and a standard deviation of 20, 86 patients are needed for this study to be included in each arm. Considering up to 20% loss to follow-up, 108 patients need to be included per treatment arm. We consider this sample size calculation to be conservative; the mean and difference estimates used were based on previous studies with PCL-5 score as the primary endpoint [61, 62]. The calculation was performed in PASS v11 [63] using an alpha of 0.05 and a power of 90.3%.

### Data management

Privacy of participants will be guaranteed by assigning a different number to each participant starting with 1 for the first patient who is included in the study. All gathered data from the participants will be stored under this number. The data being gathered consists of paper questionnaires and digital data. The key between the participant’s code and the data will be stored by the researchers in an online file which is secured with a password in a secured digital database. The data from the questionnaires are gathered using a secured, encrypted connection (https) and are stored in an online, password-protected, secured database that is only accessible by the researchers via Castor EDC. Data will be exported into separate SPSS to be used for statistical analyses.

Interview data on paper will be stored in a locked closet, in a locked room under the participant number, and only the researchers have access to the key. At the end of the study, data will be inserted in the same SPSS file as the electronic data for analyses.

Data will be kept for 20 years in accordance with national guidelines. We will submit modifications to this protocol to the approving ethical committee, the institutional review board, participants and investigators.

Due to the minimal risks of the early intervention EMDR and the short time span of the intervention, a data monitoring committee is deemed unnecessary.

### Statistical analysis

#### Primary study parameters

For the CAPS-5, an independent *t*-test will be performed to compare symptom severity scores between groups at T1. If the assumption of normality is violated, a Mann-Whitney *U* test will be performed. A linear mixed model analysis will determine the difference between the intervention group and the CAU group in changes in PTSD symptom severity as measured by the PCL-5 measured at inclusion and at follow-up (T0 and T1). Changes in scores of the PCL-5 will be modelled as a function of the intervention group (EMDR therapy, standard), time of measurement (T0–screening, T1 follow-up), and the interaction between time and intervention. Previous and current psychological treatment, educational level, parity, mode of delivery (vaginal birth, caesarean section, vacuum-assisted birth) and place of birth (at home, in the hospital under medical care, in the hospital under care or the midwife) will be entered as covariates. Per protocol analyses and intention-to-treat analyses will test the main effect of treatment condition, the main effect of time and the interaction effect. The assumptions of normality, homogeneity of variances and sphericity will be tested prior to interpreting the results. Furthermore, adverse events will be reported, and chi-square tests will be done to test differences in the prevalence of adverse events between the intervention and the control group.

#### Secondary study parameters

An independent sample *t*-test will be used to assess the differences in scores on the PBQ, W-DEQ B, WHOL-QOL and EPDS between groups on a continuous scale. Also, a chi-square test will be conducted to compare the frequencies of moderate (scores 26–40) and severe bonding disorders (scores 40 or higher) on the PBQ, the presence of FoC by using a cut-off score on the W-DEQ B of 85 or higher to indicate FoC and the differences in the prevalence of breastfeeding between groups. Linear mixed model analyses will determine the difference between the intervention group and CAU group in scores on PBQ, W-DEQ B, WHOQOL-BREF and EPDS at inclusion and at follow-up (T0 and T1). Previous psychological treatment, educational level, parity, mode of delivery and place of birth will be entered as covariates.

#### Other study parameters

Descriptive statistics will be used to evaluate demographic and clinical baseline characteristics of the arms of the trial. Chi-square tests and *t*-tests will be used to compare demographic and clinical characteristics of subjects who did not complete the intervention or follow-up with those of the completers.

### Interrater reliability and treatment fidelity

Patients, therapists and researchers will not be blinded due to the nature of the study. Assessment of the CAPS-5 interview will be conducted by an independent trained clinical interviewer, who is not aware of the randomization result. The therapists are independent of the research team and work at different sites outside the hospital. Patients in the EMDR therapy group will be randomly and equally distributed among therapists. All EMDR sessions will be recorded on video and randomly rated for treatment fidelity. During the entire study, duration group supervision every 2 to 3 months is obligatory for all therapists. After each first session with a new patient, a case conceptualization will be sent for supervision including the story of the traumatic event, with a hierarchy of the most relevant traumatic moments (targets) regarding the current impairment. Deviations from the protocol will be noted and reported. If known, therapists register when a patient terminates the study and the reason for stopping. Furthermore, they inform the PI about it as soon as possible. Women who withdraw from treatment will be invited to fill in the previously mentioned questionnaires and the CAPS interview.

### Dissemination and implementation

After completion of the study, the results will be submitted for publication to peer-reviewed scientific journals. Furthermore, results will be shared at national and international conferences, in Dutch or international publications, and possibly used for education and training purposes.

## Discussion

The PERCEIVE study will be the first randomized controlled trial that examines the safety and effectiveness of early EMDR therapy in preventing or reducing PTSD (symptoms) in women with a traumatic birth experience. We consider this of great importance given the major impact of PTSD (symptoms) on both mother and infant found in the literature [14–17]. The processing of having experienced an impactful event in relation to the birth of a child is expected to positively influence the women’s and infants’ quality of life in the longer term.

The study has several strengths. Firstly, the randomized controlled design eliminates bias in treatment assignment. All women who report having experienced giving birth as traumatic, despite the type of delivery or presence of complications and PTSD symptom severity at baseline, will be included and randomized. Hereby, the preventive effect of early intervention EMDR, as will be assessed in this study, may be generalized in all women reporting a traumatic birth experience. Another strength of this study is that PTSD (symptoms) will be assessed using a clinical interview, which will be conducted by an independent assessor 4 weeks after the last EMDR therapy session to reliably determine the presence of PTSD. To this end, it is important to note that this is the first protocol using the CAPS-5 interview in this specific population, which will add importance to the study, particularly when compared to past research which mainly used self-reporting questionnaires. Clearly, we need to test whether our assumptions are supported, but we consider it as an advantage that our treatment protocol consists of only two treatment sessions which make it easy to implement in existing care systems. Another advantage is that the study uses a broad variety of secondary outcome measures including fear of childbirth, mother-infant bonding, quality of life, breastfeeding and depression.

Yet, some limitations of the present study should also be noted. First, due to the nature of the study, patients and researchers cannot be blinded. To limit potential bias, women will be informed in which group they are allocated after finishing the first pre-treatment assessments, and the clinical interview will be conducted by an independent person. Secondly, since there is no current protocol for women experiencing traumatic childbirth, ‘usual care’ might differ individually. Therefore, a compromise has been made to give all women in the CAU group an expectative policy. They will receive two telephone calls during the study to monitor their symptom severity. When symptoms worsen significantly during the study period, the women in the CAU group will be referred to their general practitioner. Another limitation of the study protocol is the definition of the A criterion in the DSM-V for PTSD diagnosis. Not all women reporting a traumatic birth experience will be exposed to (imminent) dead, severe injury or sexual violence during the delivery, which may cause difference in PTSD diagnoses based on the course of the delivery rather than the effect of early intervention EMDR therapy itself. To limit this bias, specific questions focused on childbirth-related PTSD are added to the A criterion. Finally, because of the COVID-19 epidemic, patients as well as therapist will be at risk of being quarantined during the study period. Given the short time span in which therapy sessions have to be conducted, it is decided to exceptionally allow therapy sessions to take place digitally in these cases. Although little is known about the effectiveness of online EMDR therapy for PTSD yet, it is expected that effects may be similar to real-life EMDR therapy [64].

If early EMDR therapy proves to be effective in preventing or reducing PTSD (symptoms) after a traumatic birth experience, this would provide a strong argument for standard screening of women for traumatic experiences after giving birth and to refer them for treatment in an early phase after the delivery.

## Trial status

Protocol version 5, date of approval 10th of February 2021. Screening and recruitment started on 11 September 2020 and will continue to approximately the end of 2022.

## Data Availability

Not applicable.

## Abbreviations

CAPS-5: Clinician-Administered PTSD Scale for DSM-5
CAU: Care as usual
DSM: Diagnostic and Statistical Manual of Mental Disorders
EMDR: Eye movement desensitization and reprocessing
EPDS: Edinburgh Postnatal Depression Scale
FOC: Fear of childbirth
MIB: Mother-infant bonding
PBQ: Postpartum Bonding Questionnaire
PCL-5: The PTSD Checklist for DSM-5
PTSD: Posttraumatic stress disorder
QOL: Quality of life
TBE: Traumatic birth experience
W-DEQ B: Wijma Delivery Experience Questionnaire version B
WHOQOL-BREF: World Health Organization Quality of Life assessment
